# The experience of caregiving for children with rare musculoskeletal conditions: a qualitative study in arthrogryposis multiplex congenita

**DOI:** 10.1186/s13023-024-03224-8

**Published:** 2024-06-14

**Authors:** R. U. Elekanachi, A. Lajoie, S. Tavukcu, L. M. Snider, N. Dahan-Oliel

**Affiliations:** 1 School of Physical & Occupational Therapy, McGill University, Montreal, Canada; 2 Shriners Hospitals for Children, Montreal, Canada

**Keywords:** Qualitative, Caregiving experience, Support systems, Rare diseases, Arthrogryposis multiplex congenita

## Abstract

**Background:**

Arthrogryposis multiplex congenita (AMC) is a group of rare musculoskeletal conditions that is associated with complex healthcare needs and long-term follow up. The literature reports significant direct, indirect, and psychosocial costs for caregivers of children with neuromuscular conditions. Due to mobility limitations and frequent hospital visits, caring for a child with AMC is complex. Other challenges experienced by caregivers include financial strain, job changes, changes in interpersonal relationships and abandonment. This study was aimed at exploring the lived experience of caregivers of children with AMC.

**Methods:**

The present study is part of a larger global mixed methods study. In the initial quantitative aspect of the study, caregivers (*n* = 158) of children and youths with AMC (aged 0–21 years) responded to a cost of care survey on an electronic platform. Of the 158 participants, 13 caregivers then further consented to participate in the qualitative aspect of the study in which a 60-min semi-structured, individual interview was conducted remotely. Open-ended questions were developed to gain a deeper understanding of the direct and indirect costs of care, their impact on the caregivers' lives and the quality of the care-giving experience. Interviews were transcribed, and a coding scheme was developed drawing from both the existing literature and the content of the interviews. A deductive and inductive thematic analysis was used to analyze the qualitative data using the NVivo® qualitative data analysis software.

**Results and conclusion:**

Five themes describing the experiences of caregivers of children with AMC emerged from the analysis of the qualitative data: 1. Impact of the caregiving experience; 2. Cost of childcare; 3. Support system for care; 4. Managing and navigating care; 5. Supporting the child’s growth and development. In addition to the results of the thematic analysis, specific recommendations shared by the caregivers included the need for support groups and provision of support to youths to prepare them for adolescence. These findings will inform resource allocation, policymaking, and support services for children with rare conditions, their caregivers and families.

## Background

Caregiving for children with disabilities encompasses a range of activities necessary to provide support for their functional limitations in daily life (e.g., bathing, dressing, managing finances, shopping, providing transportation). In the United States (USA), of the 5.9 million children with severe disabilities, almost all are being cared for at home [1, 2]. However, caregiving places a significant strain on the caregiver [3, 4]. This is a critical public health issue that significantly impacts the lives of millions of individuals [5].

Caregivers can be unpaid family members, friends, or paid caregivers [3, 4]. Informal or unpaid caregivers play a vital role in providing long-term care within the home setting and contribute to the patient’s well-being from various perspectives (e.g., physical, psychological, spiritual, emotional support) [6]. While caregiving can be rewarding in many aspects, caregivers are at increased risk of experiencing negative health consequences and are twice as likely to experience chronic health problems [7], (e.g., depression, difficulty maintaining a healthy lifestyle, challenges in accessing recommended preventive healthcare services [7]). Poor caregiver health and reduced quality of care have been shown to be associated with increased hospitalizations of the child [8] or even out-of-home placement of the child [9]. According to a study by Murphy et al. (2007) [2], caregivers were concerned that, in the long term, their own deteriorating health would negatively impact their child's by threatening their ability to continue meeting their needs. The findings of this literature emphasized the importance of gaining a better understanding of the caregiving experience in the lives of children with disabilities [1].

The experience of caregiving has been studied in several pediatric conditions, including Duchenne muscular dystrophy (DMD) [10], cerebral palsy (CP) [11, 12], and osteogenesis imperfecta (OI) [13]. Research in DMD highlighted the strong association between the economic impact of caregiving, anxiety, and depression among caregivers, with their weekly hours of leisure time re-purposed to informal care [10]. Recommendations made to improve caregivers’ mental health addressed the need for depression screening and adoption of a comprehensive approach to intervention. In another early study, the economic impact on families of children with CP in Malaysia was found to be strongly influenced by annual household income, leading local policy makers to address and prioritize the socioeconomic difficulties faced by caregivers [11]. A study on the economic impact related to caregiving for children with CP in China [12] provided recommendations to policymakers in the following areas: research, preventive healthcare, treatment and rehabilitative interventions, and public financing of health care [12]. Hill et al. (2014) investigated caregiving in the context of OI, focusing on factors influencing the quality of life for these children and their families [13]. The study aimed to develop a disease-specific quality of life measure, with the goal of enhancing outcome assessment and interventions in OI care. These studies were quantitative, while Ismail et al. (2022) used a qualitative design to complement their quantitative findings, which allowed them to identify the main themes associated with the economic aspect of caregiving [11]. Another qualitative study identified factors associated with caring for a child with a disability based on interviews with parents of children with different conditions (i.e., genetic syndromes, learning, attentional, neurological, and psychiatric disorders [2]).

Rare diseases such as arthrogryposis multiplex congenita (AMC) may pose additional challenges for caregivers [14, 15]. AMC is an umbrella term used to describe a group of congenital musculoskeletal conditions characterized by joint contractures in two or more body areas that vary with respect to their distribution, severity, and impact on joint mobility and muscle strength [16]. AMC occurs in 1 in 3000 – 5000 live births [16, 17]. Causes are variable and may include genetic, parental, and environmental factors, as well as anomalies of fetal development [16]. Individuals with AMC have limited joint movement, with or without muscle weakness, in the involved body areas [17, 18]. Contractures vary in distribution and severity, do not progress to previously unaffected joints, but may change over time due to growth and treatment [16]. Spinal deformities may be present at birth or develop throughout childhood and adolescence [16]. Depending on the underlying diagnosis, other body systems (i.e., central nervous system (CNS), respiratory, gastrointestinal, and genitourinary systems) may be affected [16]. While cognition may be affected if the CNS is involved; sensation is usually intact [16]. Consequently, the impact on mobility, activities of daily living, and participation in leisure and life situations varies from complete autonomy to significant care requirements [16, 19, 20]. Given the heterogeneity of AMC, the lived experience and needs of caregivers are unique [21]. Mody et al. (2021) quantified the economic disparities associated with congenital musculoskeletal diseases worldwide from a societal perspective and reported important inequities between countries [22]. However, there is a gap in the literature on what is known about the cost of caring for a child with rare congenital musculoskeletal diseases, such as AMC. Consequently, there is a need to better understand the experience of caregiving in AMC. Identifying factors that either promote or hinder caregiving experience may help to identify barriers to healthcare and guide local policymakers in planning effective service provision to meet the needs of parents and caregivers of children with AMC.

### Objectives

The primary objective of this qualitative study was to explore the experience of caregivers of children with AMC and to identify the factors associated with facilitating or hindering the caregiving experience in AMC.

## Methods

### Study design

This study was structured around a mixed method sequential explanatory design with the overarching aim of outlining the direct, indirect, and psychosocial costs for caregivers of children with AMC. It involved the sequential collection and analysis of quantitative and qualitative data over a 6-month period (January – August 2023). In the initial quantitative aspect of the study, caregivers (*n* = 158) of children and youths with AMC (aged 0 -21 years) responded to a cost of care survey on an electronic platform. Of these, 33 agreed to be contacted for the qualitative study, and 13 consented and participated in a remote 60-minutes semi-structured, individual interview. The present study comprised the qualitative phase of the study, aiming to provide a clearer understanding of the caregivers’ experiences in AMC. By employing multiple approaches to decision-making and addressing raised issues, the results of the study offer a comprehensive analysis of the topic. The results of the quantitative portion and integration of both quantitative and qualitative results will be reported in a separate manuscript. The study reported in this paper received ethics approval from McGill University's Faculty of Medicine and Health Sciences Institutional Review Board.

### Study instrument

Building upon the results of the cost of care survey administered in the quantitative phase, a set of 10 open ended questions (Table 1) was developed for the in-depth interviews carried out in the qualitative phase of the study. These questions underwent validation by four experts from the research team (i.e., two occupational therapists, one physiotherapist and one post-doctoral fellow) to assess the face validity of the questions. A pilot study was then conducted with two caregivers of children with AMC to test the clarity of the interview questions. The interview questions then underwent back translation from English to both French and Spanish; involving an initial translation of the questions by the first author to French and Spanish using DeepL and Google Translate. The clinical research coordinator, a native French speaker, and another native Spanish speaker then back translated the interview questions as completed by the first author from French and Spanish respectively and independently to English, making any changes that might have been lost in translation. The first author reviewed the initial interview questions before translations with the back translated interview questions to ensure the accuracy of each questions.

**Table 1:** Interview questions for caregivers of children with AMC

S/N	QUESTIONS
**1.**	Having completed the survey, is there any cost you encountered when taking care of your child (ren) with AMC that was not included in the survey?
**2.**	Could you share with us any factors that may help you care for your child(ren) with AMC?
**3.**	Could you share with us any factors that may make it difficult to care for your child with AMC?
**4.**	What factor(s) do you think facilitates (makes it easier) your care / responsibilities towards your child (ren) with AMC (e.g., environment, society, economy)?
**5.**	What factor(s) do you think impede (makes it harder) your care / responsibilities towards your child (ren) with AMC? (e.g., environment, society, economy)?
**6.**	Do you have other family members with AMC or any disability?
**7.**	Does your family income cover all the services and expenses needed to care for your child(ren) with AMC?
**8.**	Is there any issue that prevents you from providing the care you want for your child with AMC? (e.g., cost of care, insurance, service coverage, governmental programs)
**9.**	What opportunities have been provided to you for caring for your child with AMC? (e.g., positive experiences, things you may have learned.)
**10.**	Is there any special support/assistance you have or are receiving from governmental associations, support groups and/or charities for your child (ren) with AMC?

The findings of the in-depth interviews complemented the quantitative findings (to be reported elsewhere), offering further insight into the factors associated with caring for a child with AMC. Specifically, the interview questions explored quality of life-related issues, sociodemographic factors, family support, unexplored costs of care, and economic impacts on the caregivers' socioeconomic and psychosocial wellbeing. Participants also completed a cost of care survey that added other dimensions (i.e., additional costs not mentioned in the quantitative phase, facilitators, obstacles to caring for their child or youth with AMC).

### Study recruitment and procedure

**Fig. 1:**
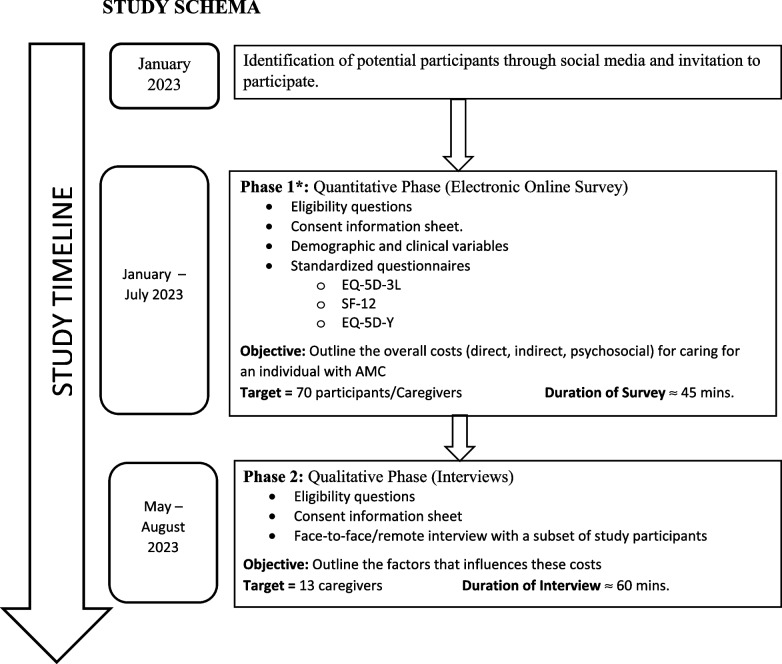
Study schema highlighting both quantitative and qualitative data collection. *Results of the quantitative phase are in review of publication

Participants were caregivers of a child or youth aged 0–21 years with AMC who had completed the quantitative phase of the study and had agreed to be contacted for the qualitative phase. The study flow is Illustrated in Fig. 1 (analysis for the quantitative phase is in progress, to be reported elsewhere). Caregivers who could communicate in English, French or Spanish, were eligible for inclusion in the study. These caregivers were approached via email by the clinical research coordinator who explained the study and obtained verbal consent. The qualitative interviews were scheduled for a duration of approximately 60 min at a time convenient to the participants and were conducted using a secure teleconferencing platform (i.e., Microsoft Teams). Caregivers were given the option to turn off their cameras if they preferred not to have their video recorded. The interviews were carried out by members of the study team (RUE, NDO). To ensure consistency, a predetermined set of interview questions was utilized during the interviews (Table 1). The audio and/or video recordings of each interview were stored in a secure research information system (i.e., Box).

## Data analysis

Given the heterogeneity of AMC, the research team ensured that participants were caregivers of children across various age groups (0 – 5, 6 – 12 and 13 – 21 years) presenting with different AMC severities, limb involvement, and mobility levels (see Table 2). The teleconferencing system provided verbatim transcriptions after each interview. Saturation was defined as repeated themes and insights identified in caregivers’ responses, and no new content identified in two consecutive interviews [21]. Saturation was reached in our data collection process during the last two interviews. Thematic analysis, a method for systematically identifying, organizing and offering insight into the patterns of meaning (i.e., themes) across a data set [23] was then used to analyze the data using a six-phase approach [23]. Phase 1: The interview transcripts were validated by a member of the research team against the video recording and stored in Box for analysis using the NVivo qualitative software. Interviews conducted in French and Spanish were also translated to English by a fluent French and Spanish-speaking member of the research team after it was validated for accuracy against the corresponding video recording. Phase 2: Initial codes for analysis (i.e., caregiver experience, worry about the future, cost of childcare) were pre-selected using a deductive approach based on existing literature that used interviews in their qualitative methodology [23, 24] on caregivers' experiences in other rare diseases [3, 10–13, 21]. The research team coded the first interview transcript after inputting the initial codes into NVivo® (Version 10). New codes were inductively added as identified and agreed upon by the team. To ensure consistency during the coding process, each domain was clearly defined (Table 3) for referral in case of doubt. Each interview underwent a coding process by two members of the research team using the agreed upon codes and every third interview transcript was coded with a third reviewer, which was consulted in case of disagreement. The coding process was followed by Phases 3, 4 and 5 (i.e., searching, reviewing, defining, and naming themes) that included summarizing themes as identified in the coded interview transcripts [25]. This resulted in the derivation of final themes and subthemes pertaining to factors associated with caring for a child living with AMC. A concurrent triangulation was done using detailed methodological and analytical steps to minimize investigator bias [21, 25].

**Table 2:** Participants’ demographic details

	**Caregiver**					**Child**			
**S/N**	**Language**	**Age**	**Country**	**Role**	**Employment status**	**Age**	**Sex**	**AMC detection**	**Limb Involvement**
1^a^	English & Spanish	46	Spain	Father	Employed, full-time	5	Male	After birth	Upper & lower limb
2	English	59	USA	Mother	Retired	19	Female	In utero	Upper & lower limb
3	English	37	USA	Mother	Employed, full-time	12	Male	After birth	Upper Limb
4	English	41	Spain	Mother	Workers’ compensation	3	Male	In utero	Upper & lower limb
5	English	43	USA	Mother	Employed, full-time	4	Female	After birth	Upper & lower limb
6	French & Spanish	35	Spain	Father	Employed, full-time	2	Male	After birth	Upper & lower limb
7	English	40	USA	Mother	Unemployed	3	Male	In utero	Upper & lower limb
8	French	-	France	Father	Employed, full-time	1	Female	After birth	Upper & lower limb
9	English	49	USA	Mother	Employed, full-time	9	Male	In utero	Upper & lower limb
10	English	57	Spain	Mother	Employed, full-time	21	Male	In utero	Lower Limb
11^a^	English	38	Canada	Mother	Unemployed	17	Female	In utero	Upper & lower limb
12	English	48	Canada	Mother	Employed, full-time	8	Female	After birth	Lower Limb
13^b^	English	54	Canada	Mother	Employed, full-time	21, 17	Females	In utero	Lower Limb

^a^Participants moved from a different country to their present country of residence (Chile to Spain; Dubai to Canada)

^b^Participant has two children with AMC and reported about both children in the interview

Four key components (credibility, transferability, dependability, and confirmability [21, 26]) were addressed to ensure trustworthiness. *Credibility* was established through data collection, analysis, and employing researcher triangulation. NVivo (Version 10), a data management tool, was used to systematically code the data and categorize specific quotes into themes [27]. Triangulation was further achieved by incorporating multiple perspectives, with some being common and others specific amongst caregivers, their socioeconomic demographics and countries into the identified themes and subthemes. A careful selection of specific quotes was done to effectively illustrate the meaning of each theme. A forward–backward translation process was employed to maintain the appropriate meaning of French and Spanish quotes when translated to English. Then, *transferability*, focused on the generalizability of the study [26, 27] was accomplished through in-depth descriptions of the themes, facilitating the application of findings to various areas of AMC and childhood disability-related care. *Dependability* was ensured through a logical, traceable, and well-documented research process [26, 27]. Next, the research design, application, data collection, and analysis were reported in detail, ensuring the *reproducibility* of the study [27]. Finally, *confirmability*, aimed to establish neutrality and minimize researcher bias, was achieved by using triangulation, detailed methodological descriptions, and the involvement of the research team in methodological (i.e., interview question development) and analytical steps, mitigating the potential impact of investigator bias [26, 28]. Phase 6 is the report of the data as seen in the Results below.

## Results

### Study subjects

Of 158 eligible participants who responded to the quantitative questionnaire, 33 participants showed interest in participating in the subsequent qualitative aspect of the study by responding ‘Yes’ to the invitation question on the quantitative survey. All interested participants were invited by the clinical research coordinator to schedule an interview with the research team. Reminders were sent every 4 weeks to ensure that every participant who indicated interest was included. Thirteen caregivers responded to the invitation to schedule an interview and provided verbal consent to participate in the qualitative study (see Table 2). Ten participants were biological mothers while three were biological fathers of children with AMC between 35 to 60 years of age (mean age = 45.41 years). Among the participants, nine were employed full time, two were unemployed, and one was retired, while one was on workers’ compensation. The age of the children (male *n* = 5, female *n* = 7) with AMC ranged from 1 to 21 years (mean age = 10.14 years). Seven caregivers reported their child’s condition was detected in utero while it was diagnosed after birth for the remaining six. Twelve of the children had no neurological involvement with AMC while it was unknown for one of the children. Nine of these children had both upper and lower limb involvement, three had lower limb involvement only and one had only upper limb involvement. Of the 13 participants, close to half were from the USA (*n* = 7) while others were living in Canada, Spain, and France.

The thematic analysis of the caregivers’ interview yielded the following five themes: 1. Impact of the caregiving experience; 2. Cost of childcare; 3. Support systems for care; 4. Managing and navigating care of the child; 5. Supporting the child’s growth and development. These themes are described below, with additional details in Table 3.

### Theme 1: Impact of the caregiving experience

This theme covered the positive and negative impacts of care on caregivers’ health and their coping mechanism including their current and future worries.

*“It is so nice to share the issues and everything, especially with other parents concerning our child.”*

By sharing their lived experiences, caregivers not only acquired valuable information but also found reassurance and gained insight into what the future holds. Caregivers mentioned improvement in their parents’ organizational and time management skills, following the gratifying effect of their child's appreciation for their efforts.

*“Taking my child, you know for the sixth time this week to a specialist appointment, so they can get XY and Z, it’s a thing and its okay and normalizing it has been my driving force and now it’s a part of everyone’s life…”*

The positive impacts extended to the entire family, as other children in the household also developed empathy towards their siblings with AMC and other children with disabilities. Alongside, caregiving has opened up opportunities, such as involvement in research and AMC support groups.

*“I am lucky to have freedom from my job because I continue to work 100%, so mostly at nights. I can be there to care for her during the day, so that is it, it helps me quite a bit indeed. ”*

Caregivers who work from home identified that the opportunity of working from home presented them with a balance, which allowed them to allocate time to both their child with AMC and their other children.

*“We were working from home, so it was easy to be there most of the time.”*

*“….Our work has made us able to organize our time, schedule and coordinate ourselves….”*

Additionally, caregivers mentioned gaining in-depth knowledge about AMC helped alleviate feelings of guilt or responsibility for causing the condition. Their better understanding of AMC is further strengthened by insights from genetic testing, reassurance that their child's condition is structural rather than due to any shortcomings on their part. However, caregiving is undoubtedly filled with challenges and presents a range of negatives that parents need to navigate. Caregivers outline that the journey of caregiving often begins with the child's birth experience, which might involve pressure to consider abortion due to the potential daunting extent of the child's condition.

*“It's very challenging, when you know the baby has AMC, of course they talk to you about abortions, you know?”*

*“It's just not what you think life is gonna be like when you get older, and no one gets pregnant and thinks they're going to have a child with disabilities.”*

The lack of proper diagnosis compounds the fears of parents, making the experience of caregiving more overwhelming. Feelings of isolation were also identified as some partners of caregivers struggle to cope, and some caregivers experienced loss of friendships due to time constraints from the child's needs.

*“After I gave birth, they took my husband to see my son and showed him our son’s difficult situation, my husband came out crying saying I don't understand anything.”*

*“It's just it's an alone world.” “She is my daily office.”*

Many parents mentioned finding themselves shouldering the responsibility alone, either due to separation or the partner's lack of engagement in the child's care. Support groups were noted as often scarce, leaving parents without the solace of connecting with families facing similar challenges. Financial stress was stated as a source of anxiety for caregivers, adding strain to the situation. The perception of society weighed heavily, as parents grapple with public scrutiny and the constant need to explain their child's condition to others. The incongruence between societal expectations of new mothers and babies and their own situation fosters feelings of inadequacy.

*“Most of the time it was like I was at a breaking point.”*

*“If you don't have the luxury of stepping down from your position to take care of your child, life would look a lot harder.”*

*“People look at my son with this curiosity and not like a child but like a sick person. It’s a refusal from society.”*

Additionally, parents found themselves reevaluating their entire lives, as caregiving takes center stage, necessitating a complete reorganization of living arrangements, work priorities, and future plans. The toll was not only emotional but also physical and mental, as caregivers battled exhaustion from the demands of care, often leading to back problems and other physical difficulties.

*“As much as I tried to get to the gym and work out and do what I needed to be able to take care of her, she got heavier and I'm getting older.”*

Worries about the child's acceptance and well-being were also on parents' minds. Accepting their child's differences from others was noted as a painful process, and parents hope for acceptance by peers and teachers. Witnessing their child undergo challenging experiences adds an extra layer of emotional burden for parents, as they struggle with the unique hardships their caregiving journey entails. Despite these numerous challenges, parents demonstrated their ability to adapt as they navigate the complexities of caregiving for their child with AMC.

*“It's tough having a child with any sort of disability because you want to protect them, but you also want them to succeed, and you can't necessarily do both at the same time.”*

### Theme 2: Cost of childcare

This theme addressed the strategies employed by parents to ensure coverage of their child’s care, and socioeconomic factors associated with these costs such as provincial or state, and federal coverages. The cost of caring for a child with AMC is complex, encompassing a wide array of sources and strategies to manage the financial impact of care. Depending on the country of residence of our participants, various sources were identified to offer coverage, such as educational institutions providing physical therapy and occupational therapy, and other specialists like child life development specialists or psychotherapists. Governmental provisions were also identified, including Social Security, Medicaid, and Medicare in the USA, along with specific state insurance programs or health plans, as mentioned by participants. Additional financial opportunities included grants, early intervention programs, and family support through shared caregiving responsibilities. Associations such as Neuromuscular Disease Foundation and Adapted Sports Federation also helped to subsidize or cover adapted sports (e.g., swimming). Shriners Hospitals for Children was largely mentioned as a provider of health care services that were completely covered with no additional costs to caregivers. Caregivers also mentioned that the cost of care was higher when the child was young, as it was typically during the early years that the child had most of their surgeries and therapies.

*“The bulk of our costs in general happened when he was younger.”*

*“When he was younger there was a huge social, direct, indirect costs...”*

Caregivers adopted diverse financial strategies to secure necessary coverage. These strategies included extensive savings and allocation of house finances as emergency funds to meet unexpected medical costs. Other strategies mentioned were sacrifices made by reducing or eliminating vacations and non-essential expenses, ensuring the child's care remained a priority.

*“So, what do we cut? Well, we're not vacationing. I mean, is vacationing a need?”*

Some caregivers consulted financial advisors to ensure the family's financial health was protected. As highlighted by caregivers, while many costs of care were covered, not all services fall under insurance coverage. Private therapies and complementary services (e.g., adapted swimming, hippotherapy) were noted to not be fully covered, depending on the country where caregivers were accessing medical care. In general, public healthcare systems were reported to fall short in terms of timeliness and comprehensiveness of the intervention. In some cases, private providers were reported to offer more comprehensive care and quicker access to necessary treatments, which resulted in more costs. Caregivers opined that navigating insurance coverages, and navigating more than one insurance policy, often remained complex with their policies imposing limitations on the number of visits, type of services and activities and coverage percentages, and presenting with inconsistencies in out-of-state and out-of-country coverage. In some cases, certain congenital conditions such as AMC were identified as not covered by insurance, and surpassing insurance limits was said to require persistent communication with healthcare professionals and insurance companies. Although some caregivers had good insurance coverage, not everyone had access to comprehensive coverage, as higher incomes resulted in less accessibility to financial aid.

*“You know because I have a good income, I cannot qualify for certain things.”*

Indirect costs (e.g., travel, accommodation, missed work for appointments) added up significantly. Adaptive equipment for schooling, personal time sacrifices, and the psychosocial toll were often not accounted for. Accessibility concerns sometimes led to moving or modifying homes, incurring additional costs. In essence, caring for a child with AMC or any childhood disability required navigating a complex web of funding sources, personal sacrifices, and strategic financial planning. The process was described as a journey marked by resourcefulness, and a continuous search for ways to provide the best possible care for these exceptional children.

### Theme 3: Support system for care

This theme covered the caregivers need for regular help with caring for their child. Support systems play an important role in the journey of caregiving for children with AMC but does come with their downsides. Family and friends were noted to provide essential emotional and practical support to caregivers with older children, partners, and ex-partners being the biggest contributors to caregiving, helping with chores, lifting, and emotional well-being. Living close to family members and having friends willing to assist was noted to ease the impact of caregiving. Caregivers mentioned that absence of proximity of family was detrimental and challenging, requiring substantial travel to get some assistance, Also, some family members, partners, or friends may not fully comprehend the condition or may be unable or unwilling to assist physically or emotionally in caregiving. Paid caregivers, such as night nurses or babysitters, were also identified to offer respite and assistance during night hours or work periods. However, finding reliable paid caregivers was identified as an ongoing struggle, as the job was demanding, and remuneration didn’t reflect the intensity of the role. The COVID-19 pandemic was said to have further exacerbated the shortage of paid caregivers.

*“There’s just no one who wants to work for that wage.”*

*“Just like we all know, COVID was kind of a different time, right? We couldn’t really have caregivers.”*

Within school and daycare settings, other supports such as child life specialists, adapted equipment, and companionship, helped promote the development of children with AMC. However, some schools were seen to shy away from offering enough support and resources due to legal concerns. AMC organizations and groups were noted to provide invaluable guidance, shared experiences, emotional support, and practical assistance through conventions, publications, financial aid, grants, and social workers. However, they sometimes struggled to accommodate the diversity within AMC conditions, and disruptions like COVID-19 hindered face to face support. Technology (e.g., the Internet, social media, and messaging platforms) helped reduce the distance and fostered connections for caregivers to find other caregivers with similar experiences.

*“At the end, we looked up AMC on the Internet and found that there's an association in Spain.”*

*“And then I guess some like Internet research, like for doctors in Spain who were specialized in like arthrogryposis.”*

Healthcare systems were identified as offering specialized professionals, therapists, surgeries, and referrals, contributing to comprehensive care. Governmental agencies also provided early intervention schemes, grants, equipment support, and financial assistance, easing financial burdens. Societal and environmental factors (e.g., public park accessibility, supportive towns, and therapy dogs) created an inclusive and welcoming atmosphere. However, depending on caregivers’ location, navigating healthcare systems remains complicated, with some healthcare professionals lacking understanding about AMC and sometimes brushing off parental concerns. Support systems play an instrumental role in shaping caregiving experience, offering crucial assistance while highlighting areas for improvement and growth.

### Theme 4—Managing and navigating care of the child

This theme addressed caregivers’ navigation and management of service which requires resourcefulness, and meticulous planning across multiple domains of life. Caregivers mentioned their need to proactively seek information, often struggling to find reliable sources about AMC and its management. The Internet, social media, books, and peer interactions became crucial resources. Caregivers who were employed as healthcare professionals mentioned the ease, they experienced due to their knowledge of disability, but many noted that generic information did not always address the specifics of AMC (e.g., the level of involvement of the child’s condition).

*“Getting information was hard because it didn’t exist, there was no web page, no Google, that said what to do for these two years.”*

*“It was hard to understand what arthrogryposis was, and we discovered what it was several weeks later.”*

Education became a centerpiece, requiring parents to align their lives around their child's school schedules. Transferring or changing schools was said to be challenging, as it involved meticulous coordination and documentation. Interactions with teachers also required clear communication to ensure the child's needs were met.

*“So, we are trying to build all of our life or trying to do everything around school for our child.”*

*“The hardest thing was just dealing with the school, so I actually put a tracking device on her because you go to these Individualized Education Programs (IEP) meetings and you're told, this is where she's gonna be.”*

*“So, we had to send all of our child’s documentation and everything to the school.”*

Navigating healthcare systems was mentioned to be a formidable task. Some caregivers had to deal with moving to new countries, which entailed dealing with new systems, doctors, and insurance. Parents often found themselves as the pivot connecting specialists, therapies, and services. Although very hard to differentiate, caregivers outlined that their personal lives were intricately intertwined with caregiving. Balancing work with caregiving was a constant struggle. Obtaining work reduction permits (e.g., Family and Medical Leave Act) remained a challenge depending on the caregiver’s job and their roles. The line between personal and professional life was blurred as parents mentioned that they needed to be available for potential emergencies. Overall, securing nursing services became essential for parents in order to sustain their work commitments. Scheduling and coordination are paramount, with parents often dividing responsibilities between themselves. Vacations were noted to require extensive planning to accommodate the child's needs. Managing and navigating the complex needs of a child with AMC entailed continuous advocacy and an agile approach to problem-solving. Parents became adept at researching, networking, and organizing, while also adapting their personal and professional lives to ensure their child received optimal care. This multifaceted effort spoke to the dedication exhibited by the parents as they struggled through the complexities of caregiving.

### Theme 5—Supporting the child’s growth and development

This theme included the strategies parents incorporated to ensure that their child was well supported in their education, recreation activities and environment. In the area of education, caregivers found themselves very involved in the organization of their child’s educational curriculum. For instance, parents wanted to ensure that their child was involved in school activities (i.e., sports) and pushed for more autonomy for their child in other activities (e.g., eating real food, walking). Advocacy for their child’s Individualized Education Plan (IEP) and adaptation of educational materials was mentioned as a key parent responsibility to ensure that the child could function in class and follow academic goals.

*“This year was the first year that I had to advocate for an IEP, he's in public school this year as opposed to a charter school.”*

*“So, they make sure he has, the proper chairs he needs to sit at a table with his peers or they make sure he has a stander in his classroom so that he can stand during circle time with his peers.”*

Parents also tried to encourage their child to get involved in extracurricular activities (e.g., sports, camping, hiking). Although offered in selected places, adapted versions of these activities were hard to come by depending on the location of the families, with a lot of planning involved. Complementing therapies such as physical therapy, occupational therapy, swimming, and hippotherapy were necessary, as reported by caregivers, to support their children’s development even though some resulted in additional out-of-pocket cost. Non-invasive therapies (e.g., massages, physical therapy), and alternative therapies (e.g., adaptive swimming, hippotherapy) were also mentioned by caregivers. Other strategies such as toys for fine motor skills, embedding therapies into daily routines (e.g., activities of daily living) and consulting a child life development specialist before every medical procedure made accessing therapies and doctors’ appointments easier.

*“Therapies became a part of life, just like breathing.”*

Some caregivers mentioned that they created “days off” as having over 4–5 doctors’ appointments in a week was overwhelming for both the caregivers and their child. Transition to adulthood was a big concern for caregivers. Although vocational rehabilitation was available, depending on the country, a lack of interest in available vocation services was observed. While parents were willing to support their child’s interests, such as blogging, the financial and time commitment (e.g., buying a computer, helping their child type, etc.) made it hard for the caregivers to support their child’s interests. Other concerns raised were questions about the future and their older child finding a partner, having sex, privacy as the child matures, and independence when the caregiver was no longer to be involved in the care of the child with AMC. Normalizing disability, letting the child do things on their own, and teaching them to be their own self-advocate were some of the strategies of the caregivers.

“Having to push him to advocate for himself instead of me advocating for him. We've learned it to be helpful, to get him to the place where he’s able to walk around himself and even go to the bathroom himself.”

“Even though the person has a disability, they want more to life than just having that disability.”

“She has limitations, but she wants to find a boyfriend that also has AMC, it is hard to find.”

## Discussion

This qualitative study is the first to explore the experience of caregivers of children with AMC, shedding light on factors that facilitate or hinder the caregiving experience in AMC at the levels of the caregiver, and society in line with literature of other childhood disabilities.

Our results identified five themes in the areas of caregiving experience, cost of childcare, support system of care, managing and navigating the child’s care, and the support of the child’s growth and development. Caring for a child with AMC involves managing numerous complex physical and cognitive needs, addressing pain, and bearing the substantial costs of care [21]. Requiring the responsibility that often necessitates a significant time commitment due to frequent medical appointments, posing challenges for caregivers to maintain a typical work schedule [21].

As exemplified in a cost study in OI, another rare musculoskeletal disorder, caregiving responsibilities were seen to have financial impacts on a caregiver’s career depending on the severity of the condition [29]. The financial aspects weighed heavily on caregivers, with the high cost of childcare necessitating additional financial aid, extra hours, and sometimes limiting access to essential healthcare services and specialized schools [11]. Navigating the caregiving landscape for children with complex needs such as CP presented significant challenges [11], and other impacts such as the high costs of equipment, accessibility devices, and cost of treatments were not covered as indirect costs in a study in OI included productivity losses due to time spent trying to access reimbursements [29].Caregiving also complicated work schedules as caregivers tend to miss work a lot and are sometimes forced to change careers or step down to lower paid positions or stop working altogether [29]. The varying demands of caregiving requiring parental adaptations are such that parents may need to halt their employment, exacerbating the financial impact and contributing to mental health issues arising from both the financial stress and the caregiving responsibilities [11, 30]. Many caregivers expressed a preference for independently caring for their children, often due to a lack of available family support, limited assistance from their social circle, financial constraints linked to paid caregiving, and a lack of confidence in paid caregivers [11]. Thus, caregivers must challenge themselves in their advocacy for early and comprehensive information about the child's condition, available resources, and treatment options to facilitate informed decision-making. Caregivers have a responsibility to seek out and share practical tips and strategies for using adaptive devices and equipment to enhance the child's participation in activities they enjoy. In addition, caregivers must educate themselves about insurance policies and their coverage, to enable better utilization of available resources and prioritize advocacy for their child's needs, and, even further, equipping them with the skills to self-advocate as they grow.

Caregiving is known to have an influence on a carer’s emotional state [31] and caring for children with disabilities involves experiences of major stressors such as feeling depressed, anxious, guilt, indecision, anger, and pain [31, 32]. Therefore, caregivers are advised to embrace self-compassion, recognizing that caregiving is a journey with its challenges, and focusing on completing tasks one step at a time. The important roles of psychosocial mediators or moderators such as social support cannot be overlooked by service providers when planning interventions for caregivers [31]. Emotional support may stem from formal and informal sources, therefore appropriate support should be provided by the respective agencies [31]. The presence and satisfaction derived from social support has been proven essential, with partner support playing a critical role in alleviating caregiver stress. A recommendation gathered from the meta-analysis of articles in a study by Almasri et al. (2018) was for service providers to engage in ongoing conversation to better understand the family's needs and to provide then with information on services, community programs, and parent support groups [30].

In addition, support programs should be designed with interested participants (i.e., caregivers and families) to help in enhancing the sense of self-perception such as self-efficacy and self-esteem, educating positive coping strategies, and building social support networks [31]. Policy makers or service providers should also strive to improve the current or existing programs in order to meet the needs of caregivers, thus reducing the long-term negative impacts on parental health and their quality of life [31]. Children with AMC may face activity limitations and participation restrictions, thus requiring substantial adaptations and persistent effort to promote independence [21]. Caregivers also undergo considerable emotional turmoil, fearing their child might miss out on education and making significant sacrifices to ensure their child's educational needs are met, including encountering difficulties in accessing inclusive programs within the school system [11]. Hence, advocacy for more accessible environments is necessary to ensure that public spaces, educational institutions, and recreational facilities are designed to accommodate children with disabilities. Our study buttressed the key role of society in increasing awareness and understanding of childhood disabilities, acknowledging the additional time and the effort caregivers invested in daily tasks due to their child's needs. Concerns regarding finding a partner and managing a fulfilling sexual life for a child with AMC are pertinent issues [21]. Hence, there is a stated need to foster more inclusive support groups catering to diverse age groups and varying levels of condition severity, thus providing a forum for sharing caregiving experiences and advice.

According to Castro and colleagues (2022), caregivers also experienced difficulty with reimbursement following the time and effort involved in caregiving [29]. Complexity in navigating the healthcare and insurance system and the unpredictability of some rare conditions made things organizationally and financially difficult. Therefore, healthcare professionals should be prepared to provide accurate and objective information about the child's condition promptly, empowering parents to plan and access necessary resources.

The significance of healthcare services that offer support, understanding, and vital information is crucial in mitigating the challenges faced by parents in caregiving roles [32]. Hence, advocacy for early detection and intervention are essential to support caregivers of children with AMC. This includes facilitating connections to case managers and other multidisciplinary teams for AMC and facilitating a comprehensive support network for caregivers. Healthcare providers should offer more resources and comprehensive information to aid both individuals with AMC and their caregivers. Additionally, addressing the lack of specific interventions and specialized knowledge about AMC within the healthcare professional community is crucial for improving care and outcomes for individuals with this condition [21].

Government support, a robust public healthcare system, and aid with transportation costs are deemed crucial in alleviating the high financial costs associated with private healthcare services, school-related expenses, and overall caregiving responsibilities [11]. Hence, it is important for the policy makers to develop policies that promote inclusivity and accessibility in public spaces, educational institutions, and healthcare facilities, ensuring a more supportive environment for children with disabilities. Investment in comprehensive support programs that cater to children of all ages and various degrees of condition involvement and promote the availability of educational and informational resources for caregivers, empowering them to provide the best care possible. Collaborating with advocacy groups to drive awareness and enhance accessibility, recognizing the significance of IEPs and prioritizing their timely implementation for children with disabilities will not only alleviate the impact of caregiving in AMC but also contribute to the overall well-being of individuals with disabilities.

This study has several limitations. Individuals worldwide were invited to participate using social media and AMC networking channels. Responses came from westernized countries and languages included English, French or Spanish. Thereby, caregivers from low and middle – income countries were not represented. Literacy levels, language, and technology barriers were also communicated to the research team as a limitation for some caregivers with children/youths with AMC in some low and middle – income countries to participate in this study. Since the recruitment for this study was mostly done using internet and social media channels, and recruitment flyers to prior participants of other studies, caregivers of children with AMC in rural areas may have been underrepresented. Finally, considering the rarity and heterogeneity of AMC, the small sample size makes it difficult for the study results to be generalizable.

## Conclusion

This study gathered the perspectives of caregivers of children with AMC on the experience of caregiving, using thematic analysis of individual interviews conducted with 13 caregivers of children and youths with AMC living in the United States, Canada, Spain, and France. In summary, healthcare professionals, policy makers and society must engage with caregivers to enhance their well-being in caregiving roles in order to create a more inclusive and supportive environment for children with AMC. All stakeholders have key roles in enhancing the quality of the lived experience of these children, caregivers, and their families. By enabling caregivers through increasing awareness, providing accurate information, and offering comprehensive resources, children with AMC are given the opportunity to thrive and lead fulfilling lives.

## Data Availability

All data generated or analyzed during this study are included in this Manuscript. However, data that support the findings of this study are not publicly available but only stored in a Shriners Hospital for Children (SHC) approved research information system, maintaining adherence with SHC policy, HIPAA requirements and local ethics requirements.

## Appendix

**Tables and Figures**

**Table 3:** Details of themes and subthemes from data analysis

Themes	Subthemes	Definition	Examples
Impact of the caregiving experience	1. Supporting factors 2. Impeding factors	This addresses the stress, positive and negative impact of care on caregivers' health and how they cope with identified stress including any other social or economic factors specific to the caregiver's experience that helps or makes caring for their child with AMC easy or difficult. Current and future worries of caregivers were also addressed as they related to their social life, interactions with friends and colleagues. Worries such as others not being able to care for their child, times when they are not capable of caring for their child and interactions when the child begins to transition from daycare to elementary school, high school and into adulthood were also included	Stress of caregiving, negative impact on caregiver health and caregiver coping strategies, caregivers’ employment status, income and partners income, type of insurance, how many jobs they have to cover their child's care; worry about the future—when caregiver won't be there anymore
Cost of childcare	1. Sources of coverage 2. Financial strategies 3. Insurance and Healthcare coverage 4. Governmental coverage	The strategies that parents implemented to ensure that the cost of their child’s care was covered, the socioeconomic factors associated with these cost such as provincial and governmental coverages. The resourcefulness they developed was documented and included how different costs reported are covered, e.g., finding funds that can cover some costs, having savings and having full insurance coverage	Financial strategies, resourcefulness for child`s care and how cost is covered
Support system for care	1) Family & close support 2) Paid caregivers 3) Healthcare support 4) Governmental support 5) Societal and environmental support 6) Other types of support	Caregivers' need for regular help with caring for their child (family, friends, church members etc.) such as breaks during school days and weekends or respite. Including when the care can and can’t be shared. Other types of support mentioned by the caregivers were also included	School support, AMC support group that linked to other families and shared information
Managing and navigating care of child	1) Personal & Knowledge acquisition 2) Education 3) Healthcare system 4) Employment	Navigation and management of services such as healthcare, and other services like education, leisure, and support systems	Satisfaction with services, and services relating to the care of their child
Supporting the child’s growth and development	1) Supporting factors 2) Impeding factors	Includes strategies parents implored to support child’s personal growth such as education, recreational and different environmental strategies to support child’s growth—not financial e.g., sport group etc	Parent seeking strategies for support education, environmental, societal

## Abbreviations

AMC: Arthrogryposis multiplex congenita.
CRC: Clinical Research Coordinator.
DMD: Duchenne muscular dystrophy.
CP: Cerebral palsy.
OI: Osteogenesis imperfecta.
USA: United States of America
